# Computer Image Analysis Reveals C-Myc as a Potential Biomarker for Discriminating between Keratoacanthoma and Cutaneous Squamous Cell Carcinoma

**DOI:** 10.1155/2022/3168503

**Published:** 2022-08-23

**Authors:** Xinyun Fan, Xueli Niu, Ze Wu, Lu Yao, Shirui Chen, Wenyu Wan, Bo Huang, Rui-Qun Qi, Tao Zhang

**Affiliations:** ^1^Key Laboratory of Immunodermatology, Ministry of Education, Department of Dermatology, The First Hospital of China Medical University, Shenyang 110001, China; ^2^Key Laboratory of Immunodermatology, National Health Commission of the People's Republic of China, The First Hospital of China Medical University, Shenyang 110001, China; ^3^National and Local Joint Engineering Research Center of Immunodermatological Theranostics, The First Hospital of China Medical University, Shenyang 110001, China; ^4^Department of Dermatology, General Hospital of Northern Theater Command, Shenyang 110000, China; ^5^Department of Pathology, Cancer Hospital of China Medical University, Liaoning Cancer Hospital & Institute, Shenyang, 110042 Liaoning Province, China; ^6^Department of Stem Cells and Regenerative Medicine, Shenyang Key Laboratory of Stem Cell and Regenerative Medicine, China Medical University, Shenyang 110122, China

## Abstract

The distinction between Keratoacanthoma (KA) and Cutaneous Squamous Cell Carcinoma (cSCC) is critical yet usually challenging to discriminate clinically and histopathologically. One approach to differentiate KA from cSCC is through assessing the immunohistochemical staining patterns of the three indicators, *β*-catenin, C-Myc, and CyclinD1, which are critical molecules that play important roles in the Wnt/*β*-catenin signaling pathway. Ki-67, as a proliferation biomarker for human tumor cells, was also assessed as an additional potential marker for differentiating KA from cSCC. In this report, these four indicators were analyzed in 42 KA and 30 cSCC cases with the use of the computer automated image analysis system. Computer automated image analysis is a time-based and cost-effective method of determining IHC staining in KA and cSCC samples. We found that C-Myc staining was predominantly localized in the nuclei of basal cells within KA patients, whereas cSCC staining was predominantly localized in the nuclei of diffuse cells. This C-Myc staining pattern has a sensitivity of 78.6% and a specificity of 66.7% for identifying KA. Moreover, positive rates of distinct expression patterns of C-Myc and Ki-67 may also serve as a means to clinically distinguish KA from cSCC. Taken together, our results suggest that these markers, in particular C-Myc, may be useful in differentiating KA from cSCC.

## 1. Introduction

Keratoacanthoma (KA) is a proliferative, fast-growing squamous epithelial skin tumor which shows a spontaneous regression. It most commonly occurs in the elderly, mainly in sun-exposed areas, with the face being particularly vulnerable, while multiforms and rash types are less common. According to the clinical manifestation of KA, the biological development of KA can be divided into proliferative, mature, and regressing stages. In all of these cases, there is a tendency to progress to a well-differentiated Cutaneous Squamous Cell Carcinoma (KA-cSCC) and KA with complete progression to cSCC [1].

KA shares many clinical and histological features with the well-differentiated cutaneous condition of Cutaneous Squamous Cell Carcinoma (cSCC). In specific, the morphological and histopathological characteristics of KA are very similar to those of cSCC, making it difficult to distinguish between these two conditions. In fact, some clinicians consider KA to be a variant of cSCC [2], which contributes to the controversy regarding the definition and diagnosis of KA. However, genomic differences, prognosis, and metastasis rates that exist between KA and cSCC suggest that these two conditions do not belong to the same category [3–5]. Moreover, reports showing a high incidence of KA (409/100,000 person-years) concentrated among residents of Queensland, Australia [6] also suggest that notable differences are present between KA and cSCC. Alternatively, such findings may likely grossly underestimate the incidence of KA due to the misclassification of these lesions as well-differentiated cSCC, underreporting, and/or spontaneous regression of KA prior to diagnosis. Given these issues, a timely and accurate differential diagnosis between these two conditions is crucial.

An important factor which can aid in such a differential diagnosis is the identification of specific, discriminatory clinicopathological biomarkers. Some clinicians have recommended that Ki-67 may possess this potential as it shows a positive percent of >20% in cSCC and ≤20% in KA [7, 8]. It has also been reported that MYC gene copy number aberrations are more common in cSCC [9, 10]. MYC genes include C-Myc, N-Myc, and L-Myc, among which C-Myc represents one of the key target genes in the Wnt/*β*-catenin signaling pathway [11]. Activation of the Wnt/*β*-catenin signaling pathway is closely associated with the development and metastasis of different tumors, including skin, colon, and breast cancers [12, 13]. In addition, in this Wnt/*β*-catenin signaling pathway, *β*-catenin is essential for controlling hair follicle formation [14–16] and the three pathological cycles of KA are inextricably linked to the cyclic growth process after hair follicle formation. These relationships have led to the hypothesis that KA originates from hair follicles and may be a follicle-associated tumor, with the spontaneous regression of KA undergoing an apoptotic process similar to that of hair follicle degeneration [17, 18]. In support of this hypothesis are findings in the chemically induced KA mouse model which show that the Wnt/*β*-catenin signaling pathway was functionally active during the growth phase of the KA tumor and that retinoic acid- (RA-) mediated Wnt inhibition was sufficient to induce spontaneous regression of KA [19].

**Figure 1:**
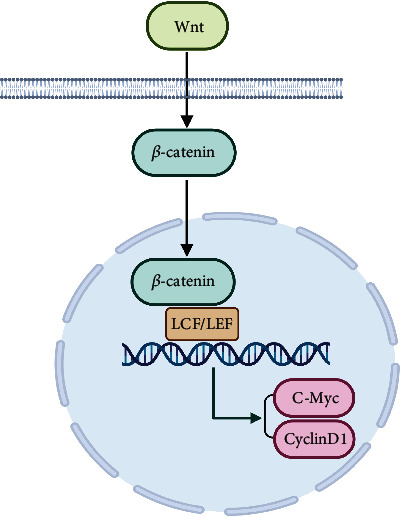
The Wnt/*β*-catenin signaling pathway.

The diverse capacities of the Wnt/*β*-catenin signaling pathway in the development of cSCC and KA led us to conjecture that key signature molecules within this pathway may play different roles and show different expression patterns in KA versus cSCC. Accordingly, it may be possible to identify and isolate specific diagnostic biomarkers to assist in the differentiation of KA from cSCC. Given this potential, we selected critical molecules within the Wnt/*β*-catenin signaling pathway cascade, including *β*-catenin, C-Myc, and CyclinD1, for analyses (Figure 1). Ki-67, being one of the most commonly used molecules in immunohistochemical studies to mark cell cycle regulation and proliferation ability [20], was also assessed. Expression patterns of these potential markers were evaluated in samples from KA and cSCC patients, using computer automated image analysis software to assess whether any differences in their protein expression profiles would be present within these two types of cancer. With these analyses, it is possible to describe the specific molecular and functional changes during tumor progression and initially explore the notion that KA and cSCC are distinct squamous epithelial proliferative skin tumors, as well as to determine whether specific molecules can be used as biomarkers for the differentiation of KA versus cSCC. Such information will be critical for the stimulation of new directions in research needed for the development of future targeted therapies.

Pathology evaluation represents the “gold standard” for tumor diagnosis. With traditional pathology assessments, the pathologist's subjective decision is based on microscopic observations, with the pathology image information stored on glass slides. Unfortunately, the accuracy involved with such procedures is not ideal and the information stored on the glass slides leads to difficulties in information processing and transfer. Moreover, the image information interpretation software currently available is relatively inconvenient. However, pathology slides can be fully digitalized with the use of whole slide imaging (WSI) technology, which utilizes the immense computing power of computers to perform computer-aided diagnosis (CAD). CAD is a technique that can quantify the shape, size, and color information of pathology images, as well as enable image retrieval, pattern recognition, computer learning, and deep learning. In this way, it is possible to construct CAD mathematical models and extract corresponding quantitative features and/or identify a specific region of interest (ROI) within a pathology image. Such quantitative features or regions obtained with CAD represent potentially important tools to assist pathologists in quantitatively evaluating indicators to achieve a more rapid, accurate, and highly reproducible medical diagnosis [21–24]. Our current experiments involving the use of inForm® automated image analysis software have indicated that this provides an accurate means to quantify biomarkers in tissue sections. Therefore, in this report, we utilized this computer analysis to identify potential biomarker differences between samples from KA and cSCC patients and, in this way, provide a more robust protocol to discriminate between these two conditions.

## 2. Methods

### 2.1. Subjects

Surgical removal of skin KA (42 cases) and well-differentiated cSCC (30 cases) samples was performed at the First Hospital of China Medical University over the period from January 2016 to May 2021. None of these patients received radiotherapy, chemotherapy, and/or biological therapy prior to surgery. All cases were assessed and confirmed independently by two pathologists. Informed consent was obtained from patients according to the Declaration of Helsinki, and the Ethics Committee of the First Hospital of China Medical University approved these experiments.

### 2.2. Histology and Immunohistochemistry

Formalin-fixed, paraffin-embedded sections were cut into 4 *μ*m sections. The sections were deparaffinized and rehydrated using xylene and graded alcohol solutions. They were blocked with 2% bovine serum albumin and incubated with specific primary antibodies for 12 hours at 4°C, followed by incubation with a biotinylated secondary antibody. Subsequently, diaminobenzidine (DAB) was added dropwise over a 3- to 5-minute period and sections were then counterstained with hematoxylin. The primary antibodies included rabbit polyclonal antibodies of CyclinD1 (dilution 1 : 200, cat. no.: ab134175, Abcam), *β*-catenin (dilution 1 : 500, cat. no.: ab32572, Abcam), Ki-67 (dilution 1 : 500, cat. no.: ab92742, Abcam), and C-Myc (cat. no.: GT220607, Gene Tech).

### 2.3. Sample Assessment

Images were generated using the Vectra™ 3.0 multiplexing image technology platform (Akoya Biosciences, DE, USA). The entire pathology section was scanned for 10x photos, then labeled using the Phenochart Whole Slide Viewer (Akoya Biosciences, DE, USA), with each tissue core scanned for production of 20x photos. The inForm® 2.4 image analysis software program (Akoya Biosciences, DE, USA) has a powerful self-learning feature enabling the identification and segmentation of specific tissue types through a “circle training” approach that allows for the measurement or scoring of biomarker expression in specific tissues, as well as within nuclear, cytoplasmic, and cytosolic regions. With this program, it is possible to convert subjective interpretational assessments of the pathology within sections into accurate and consistent objective morphological records.

**Figure 2:**
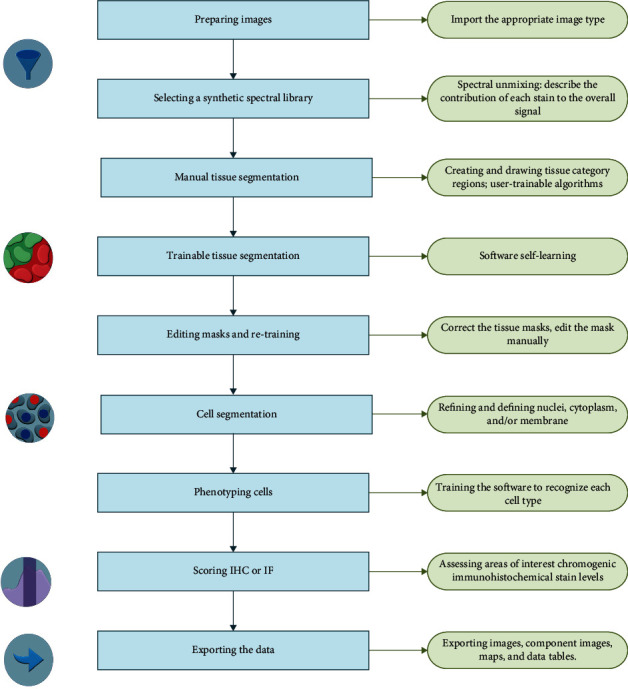
The InForm® image analysis process.

**Figure 3:**
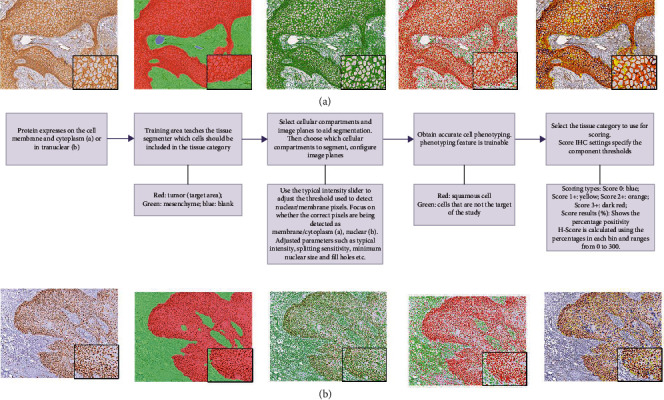
Description of the software analysis process as presented with two examples.

A brief description of the computer software analysis steps is contained in Figure 2 with *β*-catenin and Ki-67 expressions in cSCC samples presented as examples and presented in Figure 3.

### 2.4. Statistical Analysis

All data were analyzed using the SPSS 23.0 statistical software program (IBM Corporation, Armonk, NY, USA). Results are presented as the means ± standard deviations (SD). Student's *t*-tests, chi-squared tests, or one-way ANOVAs were used for these analyses. Graphs were generated using GraphPad Prism 9.0 software (GraphPad Software, Inc., San Diego, CA), Visio Drawing (Microsoft Corporation, SEA, USA), and BioRender® software (USA) with BioRender® software publication agreement number VB23ZIU9YD. A *P* < 0.05 was required for results to be considered statistically significant.

## 3. Results

### 3.1. Patient Characteristics

A summary of the demographic and clinicopathological data of patients included in this study is contained in Table 1. No statistically significant differences were obtained between the KA and cSCC patients or between the three subtypes of KA (mature KA, KA-cSCC, and KA degenerative) in terms of age, sex, and site of tumor onset (Supplemental Table 1). There was a significantly greater prevalence of KA versus cSCC at the site of sun-exposed tissue, a result that is consistent with previous findings regarding the prevalence of KA at sun-exposed sites (*P* = 0.000).

### 3.2. *β*-Catenin Expression in KA and cSCC Patients

**Figure 4:**
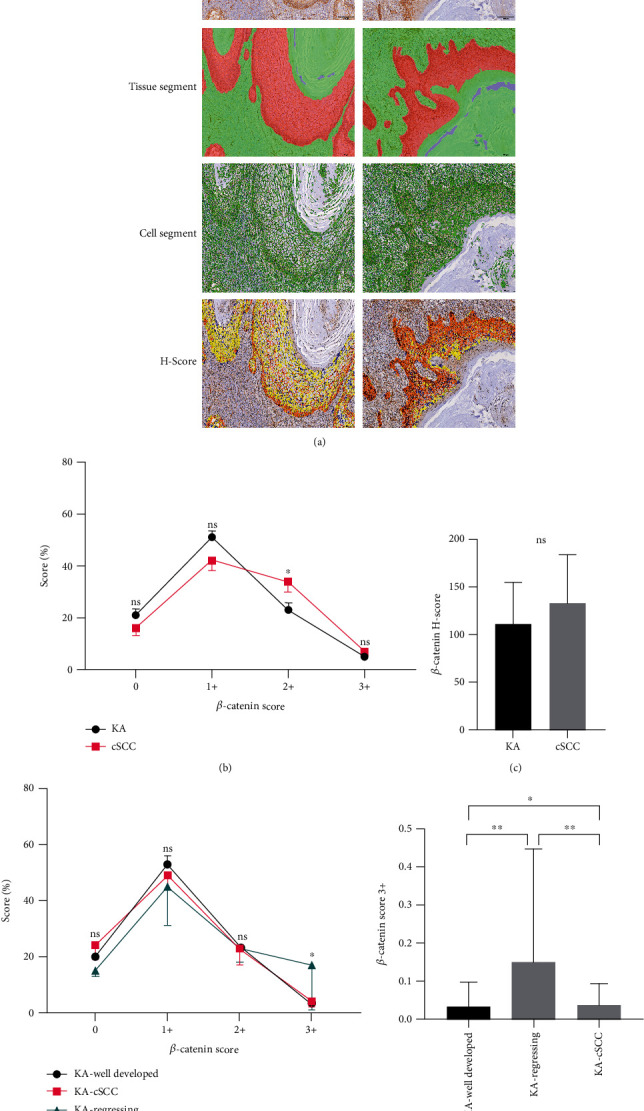
Expression and analysis of *β*-catenin in Keratoacanthoma (KA) and Cutaneous Squamous Cell Carcinoma (cSCC). (a-1) Immunohistochemical staining. *β*-Catenin in KA samples was located in cell membranes and in some portions of the cell plasma, and staining in cSCC samples revealed that it was located in cell membranes and in relatively greater portions of cell plasma (20x). (a-2) Tissue area delineation (red: tumor target area; green: mesenchyme; and blue: blank). (a-3) Cytoarchitectonic delineation (green). (a-4) *β*-catenin DAB Score delineation (Score 0: blue; Score 1+: yellow; Score 2+: orange; and Score 3+: deep red). (b) Percent of Scores 0, 1+, 2+, and 3+ in classes of KA and cSCC samples based on the intensity of *β*-catenin DAB positivity. *β*-Catenin Score 2+ (*P* = 0.026). (c) *β*-catenin *H*-Scores in KA versus cSCC samples based on intensities of *β*-catenin DAB positivity (*P* = 0.062). (d, e) Cross-comparisons of *β*-catenin Score in the three subtypes of KA (KA-well developed, KA-cSCC, KA-regressing; *β*-catenin Score 3+ *P* = 0.035); *β*-catenin Score 3+ in KA vs. KA regressing (*P* = 0.011); KA-cSCC vs. KA regressing (*P* = 0.019).

The expression of *β*-catenin in KA and cSCC patients was found to be located in cell membranes and/or the cytoplasm of the tumor island (Figure 4(a)). *β*-Catenin expression, with Score 2+, was significantly greater in cSCC versus KA patients (*P*_2+_ = 0.026, Figure 4(b)). *β*-Catenin *H*-Scores were not significantly different between the KA and cSCC samples (*P* = 0.062, Figure 4(c)). Results of *β*-catenin expression, obtained with crosstab comparisons among the three subtypes of KA, revealed that KA-regressing with Score 3+ was significantly greater than the KA-well developed and KA-cSCC subtypes (*P*_3+_ = 0.035, Figure 4(d)). Moreover, results of the within-group analysis revealed that the KA-regressing subtype was significantly greater than the KA-well developed and KA-cSCC subtypes (*P* = 0.011), while KA-cSCC, with Score 3+, was significantly greater than the KA-regressing subtype (*P* = 0.019, Figure 4(e)).

### 3.3. C-Myc Expression in KA and cSCC Patients

**Figure 5:**
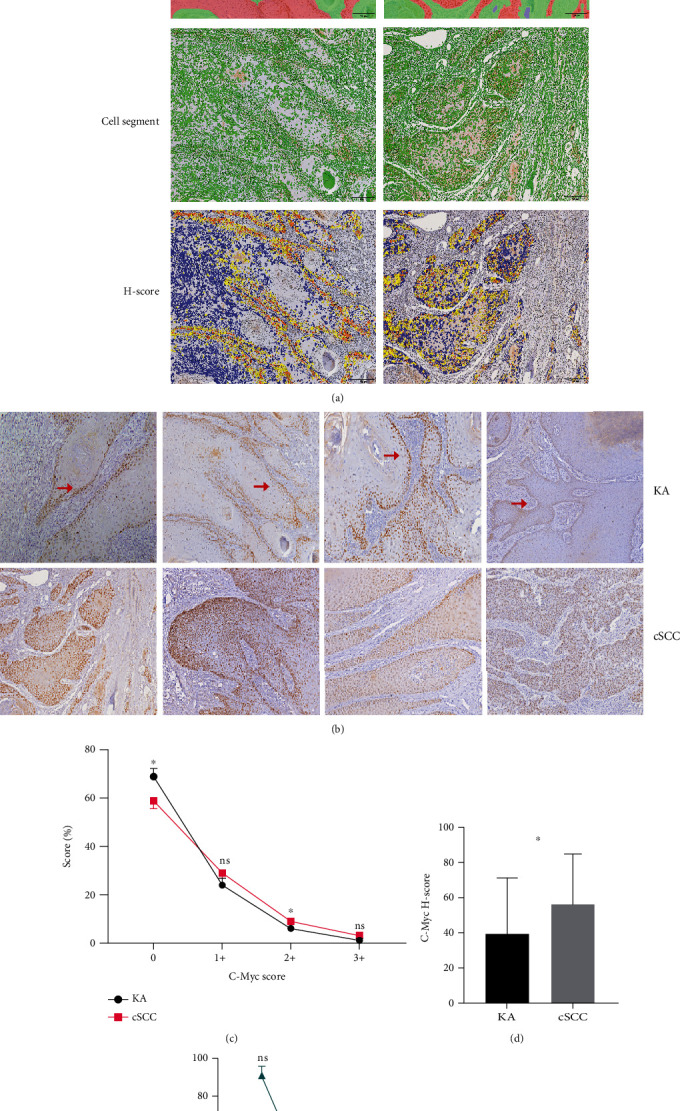
Expression and analysis of C-Myc in Keratoacanthoma (KA) and Cutaneous Squamous Cell Carcinoma (cSCC). (a-1) Immunohistochemical staining. C-Myc in KA samples was located in the initial portion of tumor infiltration, whereas in cSCC samples, there was diffuse nuclear staining (20x). (a-2) Tissue area delineation (red: tumor target area; green: mesenchyme; and blue: blank). (a-3) Cytoarchitectonic delineation (green). (a-4) C-Myc DAB Score delineation (Score 0: blue; Score 1+: yellow; Score 2+: orange; and Score 3+: dark red). (b) Expression of C-Myc in KA and cSCC patients with arrows indicating the basal region of KA tumors. (c) Percent Scores 0, 1+, 2+, and 3+ in grades of KA versus cSCC samples based on the intensity of C-Myc DAB positivity, with C-Myc Score 0 (*P* = 0.034) and C-Myc Score 2+ (*P* = 0.018). (d) C-Myc *H*-Scores in KA versus cSCC based on different intensities of C-Myc DAB positivity (*P* = 0.024). (e) Cross-comparisons of the C-Myc Score among the three subtypes of KA (KA-well developed, KA-cSCC, KA-regressing) were not statistically significant.

C-Myc expression in KA cases was mainly located in the nuclei of basal cells at the initial portion of tumor infiltration, with a few KA cases showing positive C-Myc expression within nuclei of suprabasal cells. C-Myc expression was found in the nuclei and/or cytosol of most cells in cSCC patients, with this expression being significantly greater in these regions as compared with that in other regions (Figures 5(a) and 5(b)). The C-Myc expression pattern within samples from KA patients was significantly greater than that from cSCC patients (*P* = 0.000, Table 2), with significantly increased C-Myc Scores 0 and 2+ (*P*_0_ = 0.034 and *P*_2+_ = 0.018, respectively, Figure 5(c)) and *H*-Scores (*P* = 0.024, Figure 5(d)). No statistically significant differences in C-Myc expression were obtained among the three subtypes of KA patients (Figure 5(e)).

### 3.4. CyclinD1 Expression in KA and cSCC Patients

**Figure 6:**
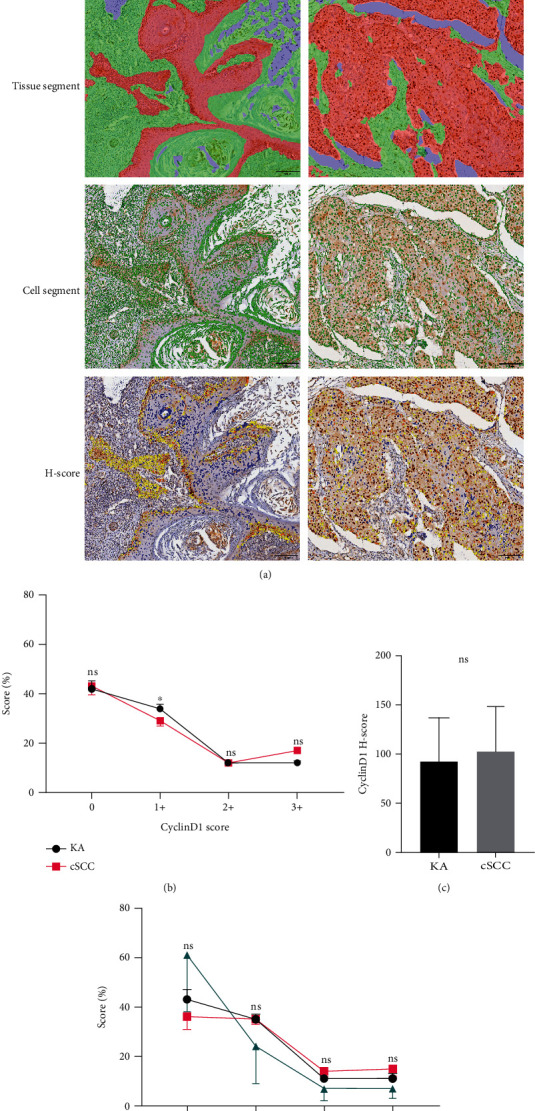
Expression and analysis of CyclinD1 in Keratoacanthoma (KA) and Cutaneous Squamous Cell Carcinoma (cSCC). (a-1) Immunohistochemical staining. CyclinD1 in KA samples was located in the initial portion of tumor infiltration, whereas in cSCC samples, there was diffuse nuclear staining (20x). (a-2) Tissue area delineation (red: tumor target area; green: mesenchyme; and blue: blank). (a-3) Cytoarchitectonic delineation (green). (a-4) CyclinD1 DAB Score delineation (Score 0: blue; Score 1+: yellow; Score 2+: orange; and Score 3+: deep red). (b) Percent of Scores 0, 1+, 2+, and 3+ in classes of KA and cSCC samples based on CyclinD1 DAB positive intensity, with CyclinD1 Score 1+ (*P* = 0.041). (c) *H*-Scores in KA versus cSCC samples based on different intensities of CyclinD1 DAB positivity (*P* = 0.360). (d) Cross-comparisons of the CyclinD1 Score among the three subtypes of KA (KA-well developed, KA-cSCC, KA-regressing) were not statistically significant.

CyclinD1 expression in KA patients showed an intense amount of positive staining within basal cells and/or portions of suprabasal cell nuclei and corresponding cytoplasm, while samples from cSCC patients showed diffuse positive staining within the nucleus and cytoplasm (Figure 6(a)). The overall expression of CyclinD1 was significantly greater in KA versus cSCC patients, with Score 1+ (*P*_1+_ = 0.041, Figure 6(b)). Results of the *H*-Score and other comparisons as performed among the three KA subtypes failed to show any statistically significant differences (Figures 6(c) and 6(d)).

### 3.5. Ki-67 Expression in KA and cSCC Patients

**Figure 7:**
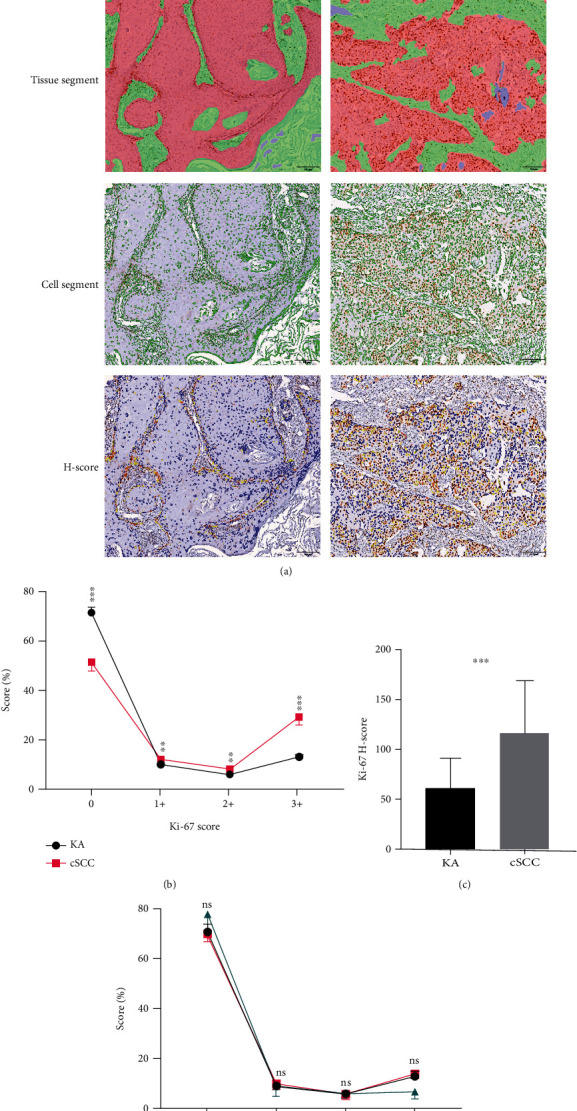
Expression and analysis of Ki-67 in Keratoacanthoma (KA) and Cutaneous Squamous Cell Carcinoma (cSCC). (a-1) Immunohistochemical staining. Ki-67 in KA samples was located in the initial portion of tumor infiltration, whereas in cSCC samples, there was diffuse nuclear staining (20x). (a-2) Tissue area delineation (red: tumor target area; green: mesenchyme; and blue: blank). (a-3) Cytoarchitectonic delineation (green). (a-4) Ki-67 DAB Score delineation (Score 0: blue; Score 1+: yellow; Score 2+: orange; and Score 3+: dark red). (b) Percent of Scores 0, 1+, 2+, and 3+ in grades of KA and cSCC samples based on differences in Ki-67 DAB positive intensities, with Ki-67 Score 0 (*P* = 0.000), Ki-67 Score 1+ (*P* = 0.011), Ki-67 Score 2+ (*P* = 0.005), and Ki-67 Score 3+ (*P* = 0.000). (c) *H*-Scores in KA and cSCC samples based on differences in intensities of Ki-67 DAB positivity (*P* = 0.000). (d) Cross-comparisons of the Ki-67 Score among the three subtypes of KA (KA-well developed, KA-cSCC, KA-regressing) were not statistically significant.

**Figure 8:**
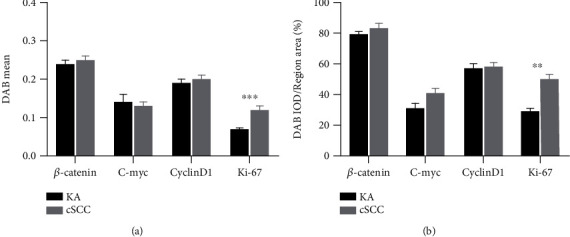
Mean and percent of DAB positivity in Keratoacanthoma (KA) and Cutaneous Squamous Cell Carcinoma (cSCC). (a) Mean of Ki-67 DAB positivity in KA and cSCC (*P* = 0.000). (b) Percent of Ki-67 positivity in KA and cSCC (DAB^+^% = Ki‐67 DAB IOD/region area) (*P* = 0.010).

Ki-67 expression within samples from KA patients was mainly localized in the nuclei of basal cells at the initial portions of tumor infiltration, with this expression being observed in the nuclei of only a few basal cells, while in cSCC patients, this Ki-67 expression was localized within the nuclei of most cells in the tumor region (Figure 7(a)). Statistically significant Ki-67 Scores 0 to 3+ (*P*_0_ = 0.000, *P*_1+_ = 0.011, *P*_2+_ = 0.005, and *P*_3+_ = 0.000, Figure 7(b)) and *H*-Scores (*P* = 0.000, Figure 7(c)) were obtained in samples from KA versus cSCC patients. No statistically significant differences were obtained among the three KA subtypes (Figure 7(e)). The mean and percent of positive Ki-67 DAB in KA samples were significantly greater than those in cSCC samples, results which were consistent with previous findings (*P* = 0.000, Figures 8(a) and 8(b)).

### 3.6. Ki-67 and C-Myc in KA Are Correlated and Consistent

**Figure 9:**
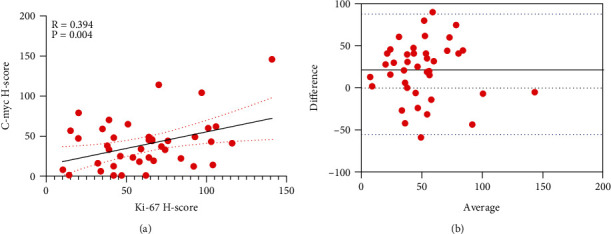
Correlation and consistency analysis of C-Myc and Ki-67 *H*-Scores in Keratoacanthoma (KA). (a) C-Myc and Ki-67 *H*-Scores showed a positive correlation (*R* = 0.394, *P* = 0.004). (b) Consistency analysis of C-Myc and Ki-67 *H*-Scores in KA (Bland-Altman plot).

The C-Myc *H*-Score was positively correlated with the Ki-67 *H*-Score in KA patients (Figures 9(a) and 9(b)). These findings of a positive correlation between Ki-67 and C-Myc prompted us to assess the diagnostic sensitivity and specificity of these two markers. We utilized the suggestion of diagnosing Ki-67 based on a positive percent of >20% as cSCC and ≤20% as KA [7, 8]. Moreover, we assigned a diagnosis of KA when the C-Myc expression mode was located in the nucleus of the basal cells at the initial portion of tumor infiltration and used an opposite criterion for cSCC. Moreover, similar diagnostic sensitivities and specificities were obtained when either tandem or parallel diagnostic methods were applied for KA and cSCC. The specificity for a diagnosis of KA with a Ki-67 of ≤20% was 96.7%. Based on the presence of the statistically significant expressions of C-Myc obtained in the KA and cSCC patients in this report, a diagnosis of KA with a specificity of 66.70% and sensitivity of 78.6% was generated when the C-Myc expression was present within the nucleus of basal cells at the initial portion of tumor infiltration. Further analysis revealed that the parallel diagnosis specificity was 66.70% and the sensitivity was 88.0%, which further improved the sensitivity of a concatenated versus a single diagnosis (Table 3).

## 4. Discussion

The results of this study reveal some noteworthy differences in the pattern of C-Myc expression between the KA and cSCC patients. To our knowledge, these findings represent the first demonstration that in samples from KA patients, C-Myc was predominantly expressed in the nuclei of basal cells located at the initial portion of tumor infiltration, along with a lower amount of expression in suprabasal cell nuclei. In contrast, C-Myc was expressed in the nuclei and/or cytoplasm in most of the cells of samples from cSCC patients. Such findings suggest that C-Myc exerts distinct activities in cSCC versus KA. Ki-67, a more established immunohistochemical biomarker of cell proliferation, provides a “tool” for quantifying tumor proliferative capacity [20]. Based on these statistical analyses performed, we found that C-Myc and Ki-67 expression patterns were correlated and consistent in KA. Interestingly, in the presence of positive Ki-67 and C-Myc expression patterns, there was an increase in the sensitivity of the diagnosis as compared with that obtained when the diagnosis was generated with separate expressions of each.

C-Myc, a critical transcriptional activator in the Wnt/*β*-catenin signaling pathway, acts as a proto-oncogene, regulating cell proliferation, differentiation, and apoptosis. The C-Myc proto-oncogene has been shown to be amplified and overexpressed in a variety of human malignancies, including head and neck cSCC [11]. With the use of a conditional transgene expression system (C-MycER™) to study the effect of conversion of C-Myc activation in different tissues of adult animals, it was found that in the mouse epidermal basal layer, C-Myc activation was sufficient to induce mitotic keratinocytes to enter the cell cycle. Sustained activation of C-Myc for 21 days results in epidermal hyperplasia with localized dysplasia, which creates papillomatous lesions resembling human precancerous skin lesions called actinic keratoses—the forerunner to cSCC. With C-MycER™ inactivation, these precancerous lesions spontaneously regress [25]. When collating these findings, it appears that C-Myc activation in the epidermal basal layer is essential for the maintenance of papillomatosis and angiogenesis and suggests a possible role of C-Myc activation and inactivation in skin tumor “reversal.” Even greater levels of C-Myc overexpression are observed in malignant cSCC. Moreover, there is evidence that C-Myc prevents keratinocytes from uncontrollable malignant proliferation and stimulates keratinocyte terminal differentiation in normal skin [26–29]. In this way, C-Myc may function as a suppressor of malignant C-Myc effects in KA by inhibiting the migration of keratinocytes to the stratum corneum, thus creating many “central corpuscles” in the tumor. Such an effect would provide an explanation for the cancerous effect of KA. C-Myc expression was concentrated in the nucleus of the basal layer at the initial portion of tumor infiltration, while in KA, this expression is observed in other areas, suggesting that the expression of C-Myc is negative. Hence, we speculate that the “multiple effects” of C-Myc can result in the spontaneous regression of KA and facilitate the transformation of KA to cSCC.

Pelengaris et al. reported that overexpression of C-Myc disrupted the differentiation of keratinocytes in the epidermis and that cSCC showed enhanced levels of proliferation and disruption of the differentiation process, results which are consistent with the concept of a “tumor MYC addiction.” MYC activation represents one of the events required for the initiation of tumorigenesis, which implies that tumor survival is dependent on high levels of MYC [30] and the hypothesis that tumor survival is dependent on increased MYC levels. Mechanistically, it appears that in tumor cells, C-Myc boosts the output of established gene expression programs, which can lead to transcriptional amplification, resulting in increased transcription levels in gene expression programs [31]. These results provide an explanation as to the means through which oncogenic C-Myc can exert differential effects on gene expression in different tumor cells and suggest that transcriptional amplification and reduced restriction of tumor cell growth and proliferation rates, along with a progressive increase in C-Myc expression, occur in highly aggressive, but poorly differentiated, tumors. Here, we demonstrate that C-Myc expression was significantly greater in cSCC versus KA.

Interestingly, analyses of C-Myc and Ki-67 *H*-Scores revealed that they were positively correlated and consistent in KA but not in cSCC. In contrast, CyclinD1, a target gene activated at comparable levels as those of C-Myc in the Wnt/*β*-catenin pathway, does not demonstrate a positive correlation with Ki-67 (*R* = 0.242, *P* = 0.095). Such results not only suggest a potentially significant effect of C-Myc but also suggest that Ki-67 and C-Myc may play similar roles in cSCC and KA. Based on previous diagnostic recommendations, we classified a Ki-67 positive percent of >20% as cSCC and that of ≤20% as KA [7, 8], along with our summary of the C-Myc expression pattern in these patients. Moreover, there is an increase in the diagnostic sensitivity with the application of two parallel diagnoses versus that obtained with a single diagnosis. In this way, the varied expression patterns of C-Myc and Ki-67 can be used to cross-reference KA and cSCC during the clinical diagnosis process, although further research with increased sample sizes will be required to substantiate these speculations. As it currently stands, Ki-67 remains one of the primary biomarkers for distinguishing KA from cSCC.

In addition to C-Myc, CyclinD1 represents another major downstream target gene of the Wnt/*β*-catenin signaling pathway. CyclinD1 regulates the cell cycle primarily by boosting the G1/S phase transition, which then permits the initiation of cell division. Under normal conditions, CyclinD1 protein is only transiently present in the cell [32], however, overexpression of CyclinD1 induces excessive skin cell proliferation and is considered necessary for the development of skin carcinogenesis, as demonstrated in a transgenic mouse model of skin carcinogenesis. CyclinD1, as one of the critical oncogenes regulating the cell cycle, disrupts cell cycle regulation in KA and cSCC, and a gradual increase in the expression of CyclinD1 can contribute to the development of a more aggressive form of the tumor [33–35]. While we found that the results of CyclinD1 expression in KA were consistent with previously published findings, the pattern of CyclinD1 expression was not significantly different between the KA and cSCC samples in the current experiment [34]. Additionally, we found that CyclinD1 expression in KA was more likely to be colocalized in the nucleus and cytoplasm of basal and multilayered spiny layer cells. Despite these findings, we believe that this does not represent a discriminatory expression pattern, suggesting that CyclinD1 expression does not play a discriminatory role in KA and cSCC.

*β*-Catenin was expressed in cell membranes and/or cytoplasm within both the KA and cSCC samples, with this expression in KA samples exhibiting a preference for cell membranes and some portions of the cell cytoplasm, while in cSCC samples, this expression was also found in the cell membrane but in relatively greater concentrations within the cytoplasm than that in KA, findings which were consistent with previous experimental results [36, 37]. The intensity of *β*-catenin expression was slightly greater in cSCC versus KA, although these differences failed to achieve statistical significance. When KA subgroups with *β*-catenin Score 3+ were compared, levels of *β*-catenin expression were significantly greater in KA-regressing as compared with the other two subtypes. We should note that these findings may be somewhat questionable due to the limited sample size of KA-regressing specimens and a dearth of research available which could explain the morphological anomalies and underlying mechanisms.

At rest, the cell has only trace levels of free *β*-catenin, while activation of the Wnt/*β*-catenin signaling pathway increases the quantity of *β*-catenin in the “cytoplasmic pool” and its translocation to the nucleus, where it initiates and regulates transcription of downstream target genes [12]. As there was an activation of the Wnt/*β*-catenin signaling pathway in both KA and cSCC in this study, the significantly increased accumulation of free-form *β*-catenin within the cytoplasm of cSCC versus KA samples implies that more *β*-catenin would be available for entry into the nucleus. As compared with KA, cSCC demonstrates an increased ability to initiate and regulate transcription of downstream target genes and promote cell proliferation and differentiation. Moreover, cSCC has a greater capacity to control and activate the transcription of downstream target genes and enhance cell proliferation and differentiation.

## 5. Conclusions

In this report, we investigated the expressions of *β*-catenin, C-Myc, and CyclinD1 in tissue samples from KA and cSCC patients. These markers represent the major molecules within the Wnt/*β*-catenin signaling pathway that are believed to be involved in the development of KA and cSCC. In addition, Ki-67, a more established biomarker, was also evaluated in these samples. To evaluate these biomarkers, we used a computerized image platform, which provides a more objective, consistent, and reproducible method for analyzing these data than that achieved with a manual review. Based on results obtained with this method, we provide the first evidence indicating that C-Myc expression in KA was predominantly located in the nuclei of basal cells, whereas this expression in cSCC samples was predominantly in the nuclei of diffuse cells. In addition, we observed that distinct expression patterns of C-Myc and Ki-67, but not CyclinD1, may serve as a means to discriminate KA from cSCC during the clinical diagnosis process. Our findings also support the notion that KA's distinctive spontaneous regression is closely tied to the Wnt/*β*-catenin pathway in mediating hair follicle development. Based on these results, we propose that KA should be considered a separate mode of skin tumor development and carcinogenesis from that of cSCC.

## Figures and Tables

**Table 1:** Summary of clinical characteristics of Keratoacanthoma (KA) and Cutaneous Squamous Cell Carcinoma (cSCC) patients.

	KA	cSCC	*P* value
Patients	42	30	
Age (years)	60.67 ± 11.29	60.97 ± 9.84	0.907
Gender (*n*, %)			0.690
Female	19 (45.24%)	15 (50.0%)	
Male	23 (54.80%)	15 (50.0%)	
Site (*n*, %)			0.000
Head and neck	35 (83.3%)	11 (36.7%)	
Body	4 (9.5%)	11 (36.7%)	
Arms and legs	39 (7.1%)	8 (26.7%)	

**Table 2:** C-Myc staining patterns in Keratoacanthoma (KA) and Cutaneous Squamous Cell Carcinoma (cSCC) patients.

	KA (*n* (%))	cSCC (*n* (%))	*P* value
Diffuse staining pattern	5 (11.90)	21 (67.74)	0.000
Peripheral staining pattern	37 (88.10)	10 (32.26)	

Note. (1) Diffuse staining pattern: C-Myc expression was mainly located in the nuclei and/or in the cytosol of most cells. (2) Peripheral staining pattern: C-Myc expression was mainly located in the nuclei of basal lamina cells at the initial portion of tumor infiltration and/or showed positive staining within the nuclei of basal lamina tumor cells and some spiny lamina cells.

**Table 3:** Comparisons of the diagnostic value of Ki-67 and C-Myc in KA patients.

	Sensitivity (%)	Specificity (%)
Ki‐67 ≤ 20%	23.80	96.70
C-Myc expression pattern 1	78.60	66.70
Tandem diagnosis	14.30	96.70
Parallel diagnosis	88.00	66.70

Note. Diagnosis 1: a positive Ki-67 fraction of ≤20% is suggestive of KA. Diagnosis 2: a positive C-Myc expression pattern in the nuclei of cells located in the initial portion of tumor infiltration is suggestive of KA. Tandem diagnosis: simultaneous combination of Diagnosis 1 and Diagnosis 2 was required for the diagnosis of KA (Diagnosis 1&Diagnosis 2 = KA). Parallel diagnosis: either Diagnosis 1 or Diagnosis 2 was required for the diagnosis of KA (Diagnosis 1 or Diagnosis 2 = KA).

## Data Availability

The data and clinical information used to support the findings of our study are available from the corresponding authors upon request.

## Supplementary Materials

Supplementary Materials Supplementary Table 1: descriptive statistics of clinical data for the three subtypes of Keratoacanthoma (KA). Supplementary Table 2: statistical description of Keratoacanthoma (KA) and Cutaneous Squamous Cell Carcinoma (cSCC). Supplementary Table 3: statistical description for the three subtypes of Keratoacanthoma (KA).
