# Supplementary material for: Computer Image Analysis Reveals C-Myc as a Potential Biomarker for Discriminating between Keratoacanthoma and Cutaneous Squamous Cell Carcinoma
Source: Biomed Res Int. 2022 Aug 23;2022:3168503. doi: 10.1155/2022/3168503 (PMC9427316; doi:10.1155/2022/3168503)
Supplement: Supplementary Materials — Supplementary Table 1: descriptive statistics of clinical data for the three subtypes of Keratoacanthoma (KA). Supplementary Table 2: statistical description of Keratoacanthoma (KA) and Cutaneous Squamous Cell Carcinoma (cSCC). Supplementary Table 3: statistical description for the three subtypes of Keratoacanthoma (KA). [file 3168503.f1.docx]

|  | **KA-well developed** | **KA-cSCC** | **KA-regressing** | **P-value** |
| --- | --- | --- | --- | --- |
| **Number** | 26 | 13 | 3 |  |
|  |  |  |  |  |
| **Age(years)** | 59.73±11.26 | 61.54±11.04 | 65.00±15.875 | 0.715 |
|  |  |  |  |  |
| **Gender(n,%)** |  |  |  | 0.722 |
| **Female** | 11（42.3%） | 6（46.2%） | 2（66.7%） |  |
| **Male** | 15（57.7%） | 7（53.8%） | 1（33.3%） |  |
|  |  |  |  |  |
| **Site(n,%)** |  |  |  | 0.311 |
| **Head and neck** | 22（84.6%） | 11（84.6%） | 2（66.7%） |  |
| **body** | 2（7.7%） | 2（15.4%） | 0（0.0%） |  |
| **arms and legs** | 2（7.7%） | 0（0.0%） | 1（33.3%） |  |

**Table 1 Descriptive statistics of clinical data for the three subtypes of Keratoacanthoma (KA)**

|  | **KA** | | **cSCC** | **P-value** |
| --- | --- | --- | --- | --- |
| **β-catenin Score(%)** |  | |  |  |
| **0** | 21±16 | | 16±16 | 0.196 |
| **1+** | 51±17 | | 42±21 | 0.054 |
| **2+** | 23±18 | | 34±23 | 0.026 |
| **3+** | 5±9 | | 7±13 | 0.321 |
|  |  | |  |  |
| **C-Myc** **Score(%)** |  | |  |  |
| **0** | 69±22 | | 59±18 | 0.034 |
| **1+** | 24±19 | | 29±12 | 0.144 |
| **2+** | 6±6 | | 9±6 | 0.018 |
| **3+** | 1±5 | | 3±4 | 0.254 |
|  |  | |  |  |
| **CyclinD1 Score(%)** |  | |  |  |
| **0** | 42±22 | | 43±19 | 0.948 |
| **1+** | 34±12 | | 29±10 | 0.041 |
| **2+** | 12±6 | | 12±5 | 0.975 |
| **3+** | 12±9 | | 17±14 | 0.063 |
|  |  | |  |  |
| **KI-67 Score(%)** |  | |  |  |
| **0** | 71±12 | | 51±19 | 0.000 |
| **1+** | 10±3 | | 12±4 | 0.011 |
| **2+** | 6±3 | | 8±4 | 0.005 |
| **3+** | 13±9 | | 29±17 | 0.000 |
|  |  | |  |  |
| **H-Score** |  | |  |  |
| **β-catenin**  **C-Myc**  **CyclinD1**  **KI-67** | 111.60±43.00  39.40±31.31  92.71±44.11  60.79±30.29 | | 132.83±51.81  56.07±28.57  102.60±45.98  115.73±52.85 | 0.062  0.024  0.360  0.000 |
|  |  | |  |  |
| **DAB Mean** |  | |  |  |
| **β-catenin** | 0.24±0.01 | | 0.25±0.01 | 0.666 |
| **C-Myc** | 0.14±0.02 | | 0.13±0.01 | 0.885 |
| **CyclinD1** | 0.19±0.01 | | 0.20±0.01 | 0.682 |
| **KI-67** | 0.07±0.00 | | 0.12±0.01 | 0.000 |
|  |  | |  |  |
| **DAB IOD/Region Area(%)** |  | |  |  |
| **β-catenin** | 79±2 | | 83±3 | 0.880 |
| **C-Myc** | 31±3 | | 41±3 | 0.185 |
| **CyclinD1** | 57±3 | | 58±3 | 0.330 |
| **KI-67** | 29±2 | | 50±3 | 0.010 |
|  | |  |  |  |

**Table 2 Statistical description of Keratoacanthoma (KA) and Cutaneous Squamous Cell Carcinoma (cSCC)**

|  | **KA-well developed** | **KA-cSCC** | **KA-regressing** | **P-value** |
| --- | --- | --- | --- | --- |
| **β-catenin Score(%)** |  |  |  |  |
| **0** | 20±15 | 24±19 | 15±3 | 0.589 |
| **1+** | 53±16 | 49±18 | 45±25 | 0.710 |
| **2+** | 24±18 | 23±21 | 23±9 | 0.977 |
| **3+** | 3±6 | 4±6 | 17±27 | 0.035 |
|  |  |  |  |  |
| **C-Myc Score(%)** |  |  |  |  |
| **0** | 67±21 | 69±23 | 91±0.09 | 0.206 |
| **1+** | 25±19 | 23±20 | 9±0.09 | 0.371 |
| **2+** | 6±6 | 7±5 | 0.00±0.00 | 0.233 |
| **3+** | 2±6 | 1±2 | 0.00±0.00 | 0.867 |
|  |  |  |  |  |
| **CyclinD1 Score(%)** |  |  |  |  |
| **0** | 43±21 | 36±18 | 61±40 | 0.184 |
| **1+** | 35±12 | 35±6 | 24±24 | 0.319 |
| **2+** | 11±5 | 14±6 | 7±9 | 0.103 |
| **3+** | 11±9 | 15±10 | 7±8 | 0.299 |
|  |  |  |  |  |
| **KI-67 Score(%)** |  |  |  |  |
| **0** | 71±13 | 70±9 | 78±15 | 0.594 |
| **1+** | 9±3 | 10±4 | 9±7 | 0.771 |
| **2+** | 6±3 | 6±3 | 6±4 | 0.716 |
| **3+** | 13±1 | 14±6 | 7±5 | 0.461 |
|  |  |  |  |  |
| **H-Score** |  |  |  |  |
| **β-catenin** | 110.34±40.23 | 106.46±46.10 | 143.00±57.17 | 0.417 |
| **C-Myc** | 42.23±33.19 | 40.69±28.19 | 9.33±9.01 | 0.227 |
| **CyclinD1** | 88.81±42.01 | 107.92±41.70 | 60.67±64.42 | 0.191 |
| **KI-67** | 61.38±33.92 | 64.08±22.15 | 41.33±28.92 | 0.508 |

**Table 3 Statistical description for the three subtypes of Keratoacanthoma (KA)**
